# Evaluating the industrial hygiene, toxicology, and public health aspects of COVID-19

**DOI:** 10.1177/0748233720964629

**Published:** 2020-09

**Authors:** Dana Hollins, Anthony L Kiorpes

**Affiliations:** 1 205740 Cardno ChemRisk, San Francisco, CA, USA; 2 Independent Consultant, Minneapolis, MN, USA

**Keywords:** Public health, toxicology, COVID, industrial hygiene, SARS-CoV-2

Since first being reported in late 2019, the outbreak of the novel coronavirus (SARS-CoV-2) has spread globally. As of the publication of this special edition, approximately 26 million cases of coronavirus disease (COVID-19) and over 800,000 of deaths have been reported in 216 countries (WHO, 2020). Research into the biology, epidemiology, bioaerosol, clinical, and other health-related aspects of SARS-CoV-2 and COVID-19 has exploded in the scientific literature in recent months, with more than 40,000 articles published in PubMed since January 2020 (PubMed, 2020). Even with this rapid dissemination of research and information regarding coronavirus, there are still gaps in our understanding of the various scientific aspects of the virus. Our aim for this special edition was to contribute to filling these data gaps in the scientific literature by devoting an entire special journal edition to scientific research related to COVID-19 in the workplace.

For nearly four decades, the journal *Toxicology and Industrial Health* (*TIH*) has been dedicated to reporting the results of basic and applied research at the intersection of toxicology and industrial hygiene with a focus on industrial/occupational health. The 12 research articles included in this special edition focus specifically on filling data gaps regarding toxicology, industrial hygiene, and public research on the current COVID-19 pandemic; these topics include application of industrial hygiene frameworks to health and safety in the post-COVID era, disinfectants, ventilation, personal protective equipment (PPE), health equity and COVID-19 risk for essential workers, and health and safety guidelines for reopening. We close the special edition with a review of viral risk mitigation strategies from the Clean 2020 virtual summit, a recent conference that aimed to assess current knowledge and identify areas for collaboration in the business, policy, science, and engineering communities regarding controlling viral transmission in the built environment. The frameworks of human health risk assessment and the hierarchy of controls in industrial hygiene guided the organization of the special edition.

The urgency of distributing timely scientific research on the coronavirus pandemic has made the preparation of the special edition challenging. We identified relevant and timely research topics (topics that may have had data gaps or that were being actively debated, such as disinfectants, ventilation, PPE/mask reuse) and reached out to active scientific researchers in these areas. However, while many researchers (such as National Institute of Occupational Safety and Health scientists or university professors) were unable to devote time to contributing to the special edition, we are grateful that so many others offered to devote their time to preparing and peer reviewing the scientific articles included in this special edition in a timely manner. The research presented in these special-edition articles is based on the data available to the authors and editors at the time of publication. We encourage readers to continually monitor international (World Health Organization), federal (Centers for Disease Control and Prevention), state, tribal, and/or local guidelines for updates and changes in recommendations, cleaning and disinfecting strategies, and other best management practices.

It is anticipated that scientific discussions concerning the science of SARS-CoV-2 and COVID-19 will continue for some time along with related discussions of how this ongoing research informs future policy decisions. We hope that the contents of the 12 papers included herein, and the extensive listing of references and resources in each paper will contribute to and facilitate continued scientific discussions and policy decisions surrounding the emergence not only of this virus but also of future outbreaks of novel diseases that impact workplace health.

## References

[bibr1-0748233720964629] PubMed (2020) PubMed search performed. Available at: search terms included “Coronavirus” OR “SARS-CoV-2” OR “Covid” OR “COVID-19” (accessed 27 August 2020).

[bibr2-0748233720964629] World Health Organization (WHO) (2020) Coronavirus disease (COVID-19) pandemic. World Health Organization. Available at: https://www.who.int/emergencies/diseases/novel-coronavirus-2019 (accessed 27 August 2020).

